# Fabrication and Biocompatibility of Electroconductive Silk Fibroin/PEDOT: PSS Composites for Corneal Epithelial Regeneration

**DOI:** 10.3390/polym12123028

**Published:** 2020-12-17

**Authors:** Promita Bhattacharjee, Mark Ahearne

**Affiliations:** 1 Trinity Centre for Biomedical Engineering, Trinity Biomedical Sciences Institute, Trinity College Dublin, University of Dublin, D02 R590 Dublin, Ireland; promitabhatt@gmail.com; 2 Department of Mechanical, Manufacturing and Biomedical Engineering, School of Engineering, Trinity College Dublin, University of Dublin, Dublin, Ireland

**Keywords:** cornea, epithelium, silk fibroin, tissue engineering, biomaterial, conductive polymer

## Abstract

The aim of this study was to develop matrices that can support human corneal epithelial cells and innervation by incorporating a conducting polymer, poly(3,4-ethylenedioxythiophene) poly(styrene sulfonate) (PEDOT:PSS), into silk fibroin (SF). Polyvinyl alcohol (PVA) was used as a crosslinking agent to enhance the mechanical properties of the matrices. The impact of PEDOT:PSS on the materials’ physical properties and cellular responses was examined. The electrical impedance of matrices decreased with increasing concentration of PEDOT:PSS suggesting improved electroconductivity. However, light transmittance also decreased with increasing PEDOT:PSS. Young’s modulus was unaffected by PEDOT:PSS but was increased by PVA. The viability of corneal epithelial cell on the matrices was unaffected by the incorporation of PEDOT:PSS except at the highest concentration tested 0.3% (*w*/*v*), which led to a cytotoxic response. These findings suggest that SF/PEDOT:PSS with a PEDOT:PSS concentration of 0.1–0.2% would be a suitable biomaterial for epithelium regeneration.

## 1. Introduction

Physical abrasions, erosion or limbal stem cell deficiencies can result in destruction of corneal epithelium and lead to blindness [1,2,3]. Biomaterial matrices may be used to culture and transplant epithelial cells to the surface of the eye, however these materials do not specifically support reinnervation. The corneal epithelium is one of the most highly innervated tissues in the human body and these nerves are essential for maintaining corneal sensation.

Nerves are vital to the cornea as they allow it to sense environmental changes and support several functionalities including blinking, tear production, wound repair and the production of trophic factors [4,5]. Nerve architecture varies with the health of corneal stroma and epithelium while being absent in the endothelium. Arising from limbal plexus and entering radially into the periphery of corneal stroma, the stromal nerve trunks contain 33–71 nerves per mm^2^ [6]. Nerves in the stroma align in parallel to the collagen fibrils that form lamellae. As these nerves progress towards the superficial stroma, they gradually split off into smaller branches. By the time they are penetrating the epithelial layer, the density has increased to 600 terminals per mm^2^ [6]. Nerve growth accompanying corneal development is affected by multiple growth factors, among which, the nerve growth factor is considered pivotal for survival, branching, elongation and regeneration of corneal nerves. Deficiency of such critical growth factors can induce neurotrophic keratopathy, leaving the cornea susceptible to injuries [6].

Silk fibroin (SF) is a one of the most studied biomaterials for corneal regeneration due to its favourable mechanical properties, optical transparency, moderate biodegradability and limited immune response upon implantation in vivo [3,6,7]. While a combination of different neuronal growth factors has been used to improve innervation into SF matrices [6,7], the combination of SF with a conductive polymer for corneal regeneration has yet to be explored. Among the different conductive polymers available poly(3,4-ethylenedioxythiophene) poly(styrene sulfonate) (PEDOT:PSS) is promising due to its excellent electrical conductivity, low reduction potential value long environmental stability, optical transparency, easy processability, its ability to be extracted by the renal system and its cytocompatibility [8,9]. Reduction potential of a polymer is a measure of its affinity towards gaining electrons and being “reduced”. This gain of electrons may take place either in the main chain or in the side chain of polymers. Measured in millivolts, the more positive the reduction potential of a polymer, the higher is its tendency to be reduced.

In the current study, SF/PEDOT:PSS based electroconductive matrices were fabricated and explored as potential biomaterials for epithelial regeneration. The combination of SF and PEDOT:PSS has not previously been explored. This specific combination led to modification of SF’s active functional groups, -NH_2_. Poly-vinyl alcohol (PVA) was used create further intermolecular hydrogen bonds, thus increasing mechanical strength of the matrix [10]. These fabricated matrices were characterized using biophysical, mechanical and in vitro biocompatibility analysis. To the best of our knowledge, SF/PEDOT:PSS composite matrix has not been utilized for corneal epithelial regeneration.

## 2. Materials and Methods

**Figure 1:**
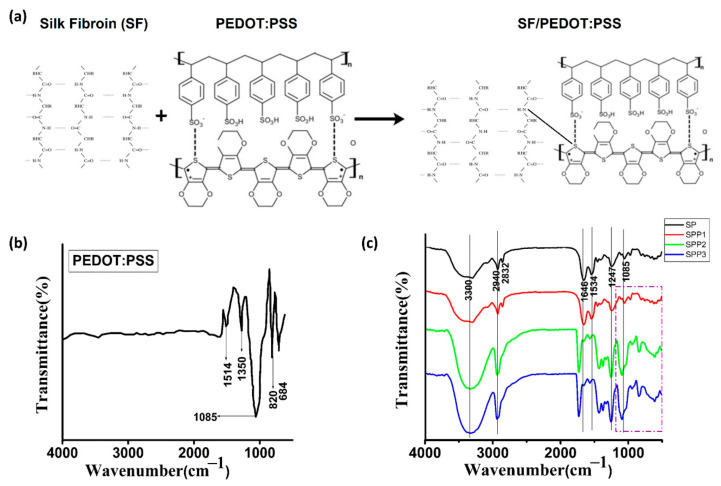
(**a**) Mechanism of fabrication of SF/PEDOT:PSS matrix; (**b**) FTIR spectra of PEDOT:PSS and (**c**) the composite matrices reveal peaks of corresponding to β sheet structure of SF and respective major absorption peaks of PEDOT:PSS without any major alterations.

When not specifically marked to be otherwise sourced, all chemicals and reagents were procured from Sigma-Aldrich. SF from Bombyx mori cocoons (Treenway Silks, Lakewood, CO, USA) was obtained using an existing extraction technique [7]. Throughout the study, 6% (*w*/*v*) SF concentration was used by continuous stirring for 8 h at room temperature, 0.1% (*w*/*v*) PVA (20,000 g/mol) solution was prepared in deionized water. Using 4:1 ratio, 6% SF and 0.1% PVA solutions were mixed for 1 h at room temperature. Following filtration through a 0.22 µm sieve, the PEDOT:PSS solution had been a final concentration of 0.892%. The PEDOT:PSS solution was sonicated (using probe sonication) for 45 min at 4 °C after filtration. Different concentrations (0.2%, 0.4% and 0.6% (*w*/*v*)) of PEDOT:PSS solution were prepared in deionized water. The PEDOT:PSS solutions were mixed with SF/PVA solutions at a 1:1 ratio and sonicated in a water-bath at 4 °C for 15 min. This resulted in PEDOT:PSS solutions of 0.1%, 0.2% and 0.3% respectively. Solutions were cast into Teflon moulds. The moulds were then vacuum dried at a temperature of 50 °C, over 24 h. To ensure β-sheet formation, the matrices were subjected to five minutes of treatment with 100% ethanol and then 20 min of treatment with 70% ethanol. The fabricated matrices were named SPP1 (0.1% PEDOT:PSS), SPP2 (0.2% PEDOT:PSS) and SPP3 (0.3% PEDOT:PSS). SF crosslinked with PVA but without PEDOT:PSS was used as a control (denoted SP). The proposed mechanism of crosslinking is presented in Figure 1a.

Light transmittance through the matrices was quantified between 300 and 700 nm using a Synergy HTX microplate reader (BioTek Instruments, Winooski, VT, USA). Matrices were soaked in 37 °C PBS for seven days prior to testing. Material composition was analysed using attenuated total reflectance-Fourier transform, infrared (ATR-FTIR, Nexus-870, Thermo Nicolet, Madison, WI, USA).

Tensile tests were conducted using a universal testing machine (UTM, Electroplus E1000, Instron, Norwood, MA, USA). The machine contained a 10 kN load cell and applied strain at a rate of 3 mm/min. Dog-bone shaped test samples (5 cm long, 1 cm wide) were prepared from the matrices and hydrated with PBS before testing. The Young’s modulus was calculated from the stress and strain data.

An Autolab potentiostat (model PGSTAT302N, Metrohm Autolab, Utrecht, The Netherlands) was used for electrochemical impedance spectroscopy (EIS) following a previously established method [8] with slight modifications to the instrument. Electrodes were connected to copper wire with silver paste and the connections were insulated with epoxy resin. After 20 h incubation in deionized water, kept at 37 °C, a 0.6 cm diameter section was taken from the swollen matrices, using a biopsy punch. This section was kept between two gold-coated glass slides—1500 nm gold coating on 15 mm × 15 mm slides. During all the measurements, the separation between the slides was kept constant at 0.7 mm using a polydimethylsiloxane (PDMS) spacer of equivalent dimension. Frequency range between 0.1 and 100 Hz, with AC amplitude of ±10 mV was used for data gathering.

Enzymatic degradation of the fabricated matrices was examined using Protease XIV. Matrices (1 cm × 1 cm) were incubated in 2 mL of enzyme solution (1 mg/mL in PBS) for up to 21 days. To estimate enzymatic degradation the residual weight of the matrices was calculated as a percentage of their original weight. The enzyme solution was changed every 2 days. Matrices in just PBS were used as a control.

Biocompatibility of the matrices was assessed using a telomerase-immortalized human corneal epithelial cell line (HCEs, Evercyte, Vienna, Austria). A previously established cell seeding protocol was followed in the current study [1]. HCEs seeded matrices (3000 cells/cm^2^) were cultured in serum-free Keratinocyte Growth Medium 2 (KGM-2, PromoCell, Heidelberg, Germany). Metabolic activity of seeded HCEs was monitored using a PrestoBlue assay (Thermo-Fisher Scientific, Waltham, MA, USA). Cell viability was examined using a live-dead assay (Molecular Probes, Eugene, OR, USA) after five days of culture. Both assays used the protocols specified by the respective manufacturers.

To visualize the arrangement of filamentous actin in cells on the matrices, cells were fixed using 4% paraformaldehyde for 15 min. Phalloidin-TRITC and 4′,6-diamidino-2-phenylindole (DAPI) were used to respectively stain actin and nuclei using a previously established protocol [1]. Images were obtained using a confocal microscopy (Leica SP8, Leica, Wetzlar, Germany) and LAS X Advanced software was used for image processing.

Data has been presented as the average value ± standard deviation, with *n* = 3, unless specified to be otherwise in specific cases. All statistical analysis used the R statistical platform. Comparisons across different matrix compositions of their mechanical properties, biocompatibility, etc., used a one-way ANOVA and post-hoc Tukey’s test. *** *p* < 0.001; ** *p* < 0.01; * *p* < 0.05 was used to mark significant differences.

## 3. Results and Discussion

The chemical modification of the functional groups (–NH_2_) of silk fibroin was exhibited after blending with PEDOT:PSS (Figure 1a). The average thickness of fabricated matrices was 258 ± 5.2 µm. It is reported that such low thickness matrices exhibit a major contribution to the substrate and oxygen crossover [11]. ATR-FTIR spectroscopy analysis (Figure 1b,c) of the fabricates showed the following peaks: 1514.17 cm^−1^, asymmetric stretching of carbon double bond in PEDOT:PSS due to altered benzoid/quinonoid structure of PEDOT:PSS thiopene ring; 1350.82 cm^−1^, symmetric/asymmetric stretching of carbon single bond in the thiophene ring; 820.30 cm^−1^ and 684.25 cm^−1^, vibration modes for the –C–S–C– bond of the thiophene ring and 1085.65 cm^−1^, bending modes of –C–O–C– bond in the ethylenedioxy group. For none of the composite matrices (SPP1, SPP2 and SPP3), significant peak shifts or transformations were observed while spectra of all three matrices contained the primary absorption peaks related to SP and PEDOT:PSS. Our results support the findings by Khan et al. [10]. Minor peak shifting was observed and marked in the FTIR image with line shifting markers. Higher peak intensity of major characteristics peaks associated with PEDOT: PSS at 1085 cm^−1^ and 820 cm^−1^ was recorded for the SPP2 and SPP3 matrices when compared with the SPP1 matrix (denoted by dotted box in FTIR spectra). This may be also due to the overlapping of two major characteristics peaks of PEDOT:PSS and SP at 1085 cm^−1^.

**Figure 2:**
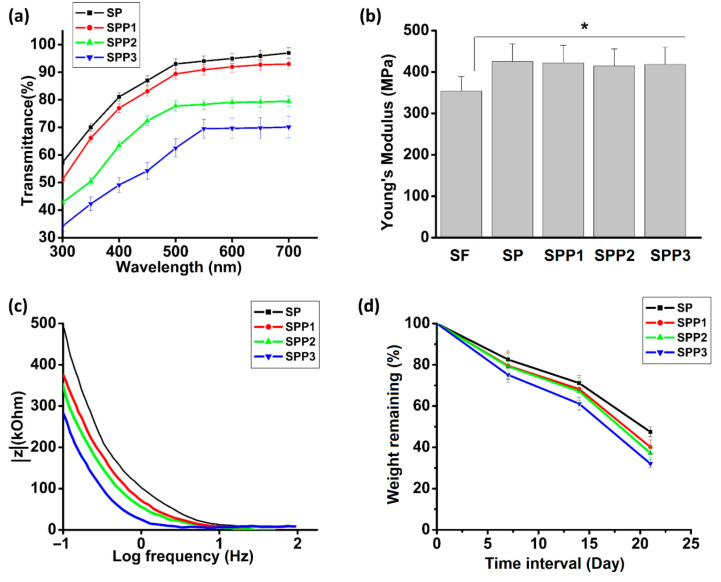
(**a**) Light transmittance and (**b**) Young’s modulus of different SF/PEDOT:PSS matrices. A significant difference (* *p* < 0.05) in Young’s modulus (MPa) observed due to incorporation of PVA. (**c**) EIS (electrochemical impedance spectroscopy) measurements of fabricated matrices containing various concentrations of PEDOT:PSS. (**d**) The degradation of matrices at 37 °C in Proteinase K over 21 days.

To maintain a patients vision, it is vital that light be allowed pass through the matrices. Visible light transmittance through the matrices was between 70% and 95% (Figure 2a). Transmittance reduced for wavelengths in the UV spectrum. These results are similar to the light transmittance found in real cornea [2]. Light transmittance reduced with increasing concentrations of PEDOT:PSS.

The effect of PVA crosslinking and PEDOT:PSS concentration on the matrices Young’s modulus was examined (Figure 2b). PVA led to a significant increase in modulus when compared with a SF matrix without PVA (denoted as SF). This is due to interfibrillar crosslinking induced by PVA. There was no significant difference in the modulus due to the addition of PEDOT:PSS.

The matrices are susceptible to high frequency capacitive currents. However, for electroactive biological tissues (1 Hz), resistive currents predominate. The electrical conductivity of the matrices was analysed using low impedance, high frequency currents (Figure 2c). SPP1, SPP2 and SPP3 matrices possessed lower impedances than SP matrices due to the presence of PEDOT:PSS. At a frequency of log(1) Hz, the impedance decreased from 479.1 kOhm for SP to 371.3 kOhm for SPP1, 345.3 kOhm for SPP2 and 275.5 kOhm for SPP3. An increase in PEDOT:PSS concentration of the matrices could be related to an increase in their electroconductivity as well.

To examine degradation resistance, matrices were treated with proteinase K for 21 days. This resulted in a time dependent decrease in weight for all matrices (Figure 2d). PEDOT:PSS led to a small increase in degradation. When submerged in the just PBS for 21 days, the weight of the matrices remained unchanged. When it comes to biodegradation of scaffolds, being able to reach a suitable degradation rate is the key. While materials that degrade too fast are not useful, a moderate rate of degradation allows the constructs to be remodelled. Vital cellular functions such as viability, migration and host–tissue response are significantly influenced by the rate of biodegradation. The suitable rate of biodegradation, in vivo, should match the rate of formation of new tissue. Silk based fabricates have been shown to have a similar degradation profile when using proteinase K as they have in vivo, due to human enzymes [12].

**Figure 3:**
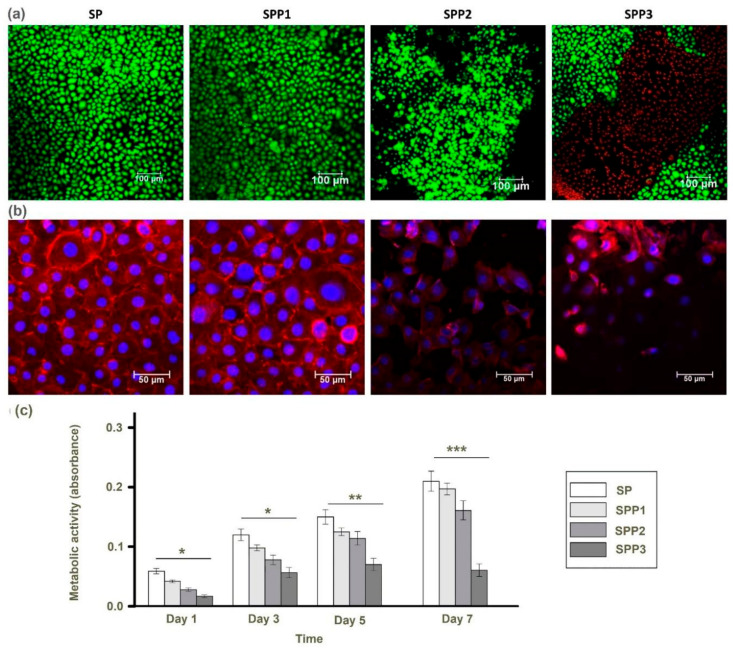
(**a**) Viability of cells on different matrices (live cells appear green and dead cells red) Scale bar = 100 μm. (**b**) Cytoskeletal organization of corneal epithelial cells after 7 days in culture after staining actin filaments (red) and nuclei (blue). Scale bar = 50 μm. (**c**) Total cell metabolism on different composite matrices evaluated by the Presto-blue assay (* *p* < 0.05, ** *p* < 0.01, *** *p* < 0.001).

Metabolic activity was used an indicator of cell proliferation. Metabolic activity increased over 7 days significantly for all matrix compositions, except SPP3 (Figure 3c). The low metabolic activity on SPP3 matrices indicated that the material was cytotoxic. This was confirmed via live/dead straining matrices after 5 days of culture, which showed large populations of dead cell present on SPP3 matrices (Figure 3a). No dead cells were present on the other matrices.

Cell cytoskeleton organization impacts cellular migration, cell adhesion and contractility of the matrix. Cytoskeletal staining (Figure 3b) of filamentous actin is a useful method of assessing cell shape and behaviour. Cells on SP and SPP1 matrices had similar morphologies with cells packed together forming an epithelial barrier. Cells on SPP2 were more spread with less cortical actin present. Cells on SPP3 were fewer in number, lacked clear actin fibres and displayed a clearly different morphology. This may be due to the cytotoxic effect of PEDOT:PSS at high concentrations.

## 4. Conclusions

The addition of low concentrations (0.1–0.2%) of PEDOT:PSS to SF was shown to improve the electroconductivity of the biopolymer while having only a minimal effect on other material properties and cell behaviour. Higher concentrations (0.3%) of PEDOT:PSS led to a cytotoxic response to the material and should be avoided. While further study is required to determine if these matrices can improve the restoration of nerves in vivo, their potential for corneal epithelium regeneration was demonstrated.
